# Associations between Chronic Community Noise Exposure and Blood Pressure at Rest and during Acute Noise and Non-Noise Stressors among Urban School Children in India

**DOI:** 10.3390/ijerph7093457

**Published:** 2010-09-15

**Authors:** Stephen J. Lepore, Bhaskar Shejwal, Bang Hyun Kim, Gary W. Evans

**Affiliations:** 1 Department of Public Health, Temple University, 957 Ritter Annex, 1301 Cecil B. Moore Ave, Philadelphia, PA 19122, USA; E-Mail: bkim@temple.edu (B.H.K.); 2 Department of Psychology, Pune University, Arts Faculty Building, Ganeshkhind, Pune 411007, India; E-Mail: brs@unipune.ernet.in (B.S.); 3 Department of Design & Environmental Analysis, Cornell University, M Van Rensselaer Hall, Ithaca, NY 14853, USA; E-Mail: gwe1@cornell.edu (G.W.E.)

**Keywords:** noise, blood pressure, stress, reactivity, habituation

## Abstract

The present study builds on prior research that has examined the association between children’s chronic exposure to community noise and resting blood pressure and blood pressure dysregulation during exposure to acute stressors. A novel contribution of the study is that it examines how chronic noise exposure relates to blood pressure responses during exposure to both noise and non-noise acute stressors. The acute noise stressor was recorded street noise and the non-noise stressor was mental arithmetic. The sample consisted of 189 3rd and 6th grade children (51.9% percent boys; 52.9% 3rd graders) from a noisy (n = 95) or relatively quiet (n = 94) public school in the city of Pune, India. There were no statistically significant differences between chronic noise levels and resting blood pressure levels. However, relative to quiet-school children, noisy-school children had significantly lower increases in blood pressure when exposed to either an acute noise or non-noise stressor. This finding suggests that chronic noise exposure may result in hypo-reactivity to a variety of stressors and not just habituation to noise stressors.

## 1. Introduction

Many children throughout the world, but especially those in developing countries, are chronically exposed to high levels of community noise [1]. Chronic noise exposure among youth usually comes from transportation (*i.e.*, road traffic, airplanes, trains), music, and other people [2]. Stress theory has been applied to help explain a variety of ill effects attributed to chronic noise exposure [1,3,4], including dysregulation of blood pressure [4–6]. In the present study, we examine how chronic noise exposure relates to children’s resting blood pressure and the regulation of blood pressure responses to acute stressors. To the extent that chronic noise exposure in children is associated with higher than average levels of blood pressure at rest and during stressors, it could be a risk factor for cardiovascular and coronary heart disease. For example, elevated resting and stress-reactive blood pressure in children has been positively correlated with elevated resting blood pressure in later years [7–9]. To the extent that chronic noise exposure has the opposite effect of diminishing children’s level of arousal in response to acute stressors, it could undermine adaptive behavioral coping responses and other biological processes relevant to health and disease processes [10].

There have been a number of studies on the link between chronic noise exposure and resting blood pressure in children (for reviews, see [1,2]). Some investigators have found higher resting diastolic blood pressure (DBP) and/or systolic blood pressure (SBP) among children exposed to high versus relatively low levels of chronic noise from automobile traffic, airplanes, or trains [3,4,11–14]. In contrast, other investigators have found gender-specific relations [15], nil relations [16], and negative relations between chronic noise exposure due to airplane or traffic noise and resting blood pressure in children [17]. Additional research will further advance our understanding of the parameters under which chronic noise may be a risk factor for elevated resting blood pressure in children.

We know practically nothing about the relation between chronic noise exposure and blood pressure reactivity during acute stress exposures in children. A novel contribution of the present study is that it examines how chronic noise stress exposure in children relates to their blood pressure reactivity when they are exposed to a stressor that matches the one they are exposed to chronically (an acute noise stressor) versus one that is more novel (an acute mental arithmetic challenge). Prior research has shown that animals repeatedly exposed to a stressor will exhibit a diminished response (habituation) when they encounter a similar acute stressor and an exaggerated response (sensitization) when they encounter a novel acute stressor [18]. Whether humans exposed to chronic stressors develop this dual response pattern of habituation and sensitization in blood pressure responses to similar versus novel acute stressors, respectively, has yet to be established.

Habituation to noise may be adaptive to the extent that it reduces levels of sustained arousal—and associated risks of such arousal—among individuals chronically noise exposed. However, a pattern of habituation to noise and sensitization to non-noise stressors might be indicative of dysregulation of the stress response system. Similarly, a pattern of diminished response to both noise and non-noise stressors might be indicative of dysregulation of the stress response system. Impairment of blood pressure responses resulting from iterative stress exposures may reflect damage to normal, healthy physiological stress-response systems that are designed to mobilize energy to support coping efforts when an acute environmental demand is encountered [19–21].

We could find only one study on the relation between chronic noise exposure and blood pressure reactivity. In that study, children in a noisy community near a major airport had lower SBP reactivity in response to an acute stressor, which consisted of intermittent noise (80 dBA peaks) while reading a text, than did their counterparts in a quieter community [22]. There was no relation between noise exposure and DBP reactivity. This study provides some evidence that children in the noisy schools habituated to noise. However, because there was no assessment of reactivity to non-noise stressors, it is not clear if there were other adaptive costs of noise exposure, such as sensitization to non-noise stressors or possible diminished arousal in response to varied stressors. The present study was designed to characterize these distinct patterns of relations between chronic noise exposure and reactivity.

In summary, the present study adds to the literature on the association between chronic exposure to community noise and levels of resting blood pressure in children. The study also examines whether chronic noise exposure compromises children’s stress response systems, by examining the relations between chronic noise exposure and blood pressure responses during exposure to acute stressors similar to the chronic stressor (acute noise) and a novel psychosocial stressor (mental arithmetic). A novel contribution of the study is that it examines the relations between chronic noise exposure and children’s blood pressure reactivity during acute exposure to both noise and non-noise stressors. The resulting data could reveal a variety of patterns, including a potentially adaptive pattern, in which children selectively habituate to noise stressors but respond normally to non-noise stressors, or a potentially maladaptive pattern, in which children habituate to noise stressors but are hyper- or hypo-reactive to non-noise stressors.

## 2. Methods

### 2.1. Setting

Participants were drawn from two public schools in Pune, India, the eighth largest city in India, with a population of over 3.7 million people [23]. The “noisy” school selected for this study was located in a busy commercial district on a street with much traffic noise. Traffic noise is probably the most pervasive type of chronic noise exposure for children, especially in India [24]. The “quiet” school was located in a less commercial area of the city and offset from the street by at least 100 yards. The buildings of the two schools are of similar construction and have classrooms with open-air windows, so outside ambient noise easily drifts into the buildings. Noise levels at both schools were measured mid-morning between the outside entrance to the buildings and the school gate. Over a 5-minute interval, noise levels at the noisy school reached a peak of 82 dB(A) and at the quiet school reached a peak of 65 dB(A).

### 2.2. Participants

A total of 189 boys and girls from 3rd and 6th grade classrooms participated in this study: 95 from the quiet school and 94 from the noisy school. Sex of child was distributed fairly evenly between the noisy school (51.6% males) and quiet school (52.1% males). Class level also was distributed fairly evenly between the noisy school (52.5% third graders) and quiet school (55.3% third graders).

### 2.3. Experimental Design

The study used a non-equivalent, two-group (quiet *vs.* noisy school) repeated-measures (noise challenge reactivity *vs.* math challenge reactivity) research design. We used a counterbalanced design to control for order effects, with children randomly assigned to order of presentation of the acute stressors.

### 2.4. Measures

*Resting and reactive blood pressure*. Blood pressure was measured using a non-invasive ambulatory blood pressure monitoring device manufactured by Finapress Medical Systems (Amsterdam, The Netherlands). The device was applied to the middle finger of the child’s non-dominant hand. Three readings were taken at 1-minute intervals. The first reading allowed the child to adapt to the device. The average of the last two readings was used to create the resting SBP and resting DBP scores. Blood pressure also was measured three times at one-minute intervals during the acute stressors, a noise challenge and a math challenge, which are described below. The reactivity scores were calculated by subtracting the average resting blood pressure reading from the average of the three blood pressure readings taken during each challenge (e.g., math challenge reactivity on SBP = mean math challenge SBP—resting SBP).

*Body Mass Index (BMI).* BMI was measured using the standard metric formula: weight in kilograms/height in meters squared (km/m^2^).

*Rating of testing environment*. At the end of each study session with a child, experimenters recorded their overall impressions of the testing environment using a 4-point scale: 0 = no problems, quiet, no interruptions; 1 = minor problems, occasional background noise and/or an interruption; 2 = moderate problems, low background noise throughout testing and/or two interruptions; 3 = major problems, high levels of background noise throughout testing period and/or more than two interruptions.

### 2.5. Procedures

The study procedures were reviewed and approved by the appropriate institutional review boards and informed consent was obtained from participating study schools. Each child was assessed individually under standardized conditions at the schools. The child sat at a desk across from the experimenter. The child’s height (meters) and weight (kilograms) were measured, a brief survey was completed to collect demographic data (e.g., age), resting blood pressure was assessed, and reactive blood pressure was assessed while the child was exposed to a noise challenge and a non-noise challenge. There was a three-minute resting period between the challenges. The acute non-noise stressor was a math challenge that required participants to subtract aloud by four from a large number and keep subtracting by four until they were told to stop at the three-minute mark. The acute noise stressor was a challenge that involved listening via headphones to a three-minute recorded mix of loud street noises, office machinery, and people speaking, with intermittent random noise bursts (80 dBA peaks) [25].

## 3. Results

### 3.1. Identification of Covariates

Preliminary analyses were conducted to identify covariates. Relative to children in the quiet school, children in the noisy school had a significantly higher BMI (20.66 *vs.* 30.97 mean BMI) (t_187_ = 11.05, p = 0.000) and were trending toward being older (9.21 *vs.* 9.67 mean years old) (t_187_ =1.87, p = 0.06). In addition, the experimenters reported slightly more problems due to noise and/or interruptions in the testing environment of the noisy school (mean = 1.22) than the quiet school (mean = 0.71) (t_187_ = 3.61, p = 0.000). We also identified sex of the child as a covariate because BMI was significantly higher in girls (mean = 27.44) than boys (mean = 24.26) (t_187_ = 2.69, p = 0.008) and DBP reactivity during the math challenge was trending toward being higher in girls (mean = 6.58 mm HG change) than boys (mean = 3.20 mm HG change) (t_187_ = 1.72, p = 0.09). Thus, sex, age, BMI and level of testing problems were used as covariates in subsequent analyses.

### 3.2. Resting Blood Pressure

Resting SBP and DBP outcomes were analyzed using separate ANCOVAs. School (noisy *vs.* quiet) was the independent variable and sex, age, BMI, and testing problems were covariates. As shown in Table 1, on average children in the noisy school tended to have lower blood pressure than children in the quiet school; but the differences were statistically non-significant.

### 3.3. Blood Pressure Reactivity during Acute Noise and Non-Noise Stressor Exposure

**Figure 1.**
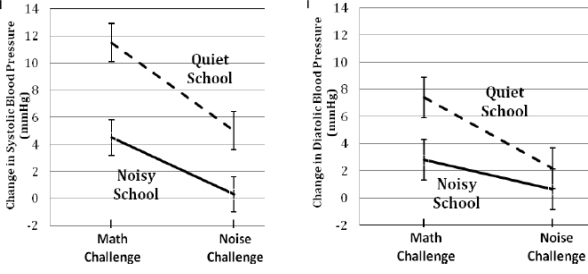
Systolic ^1^ and diastolic ^2^ blood pressure reactivity during acute math and noise stressors among children in the quiet versus noisy elementary school (n = 189). ^1^ Means adjusted for sex, age, BMI, testing problems, and resting systolic blood pressure. ^2^ Means adjusted for sex, age, BMI, testing problems, and resting diastolic blood pressure.

SBP and DBP reactivity outcomes were analyzed using separate ANCOVAs with school (noisy *vs.* quite) as the between-subjects factor and type of acute stressor (noise *vs.* math challenge) as the within-subjects factor. Each ANCOVA statistically adjusted for the corresponding baseline value of resting blood pressure (e.g., resting SBP was covaried in analyses of SBP reactivity), in addition to sex, age, BMI, and testing problems. There was no effect of order of presentation of challenge, so analyses collapsed across order. Analyses of SBP reactivity revealed a significant main effect of school (F_1,182_ = 8.73, p = 0.004), no significant main effect of type of acute stressor (F_1,182_ = 2.77, p = 0.098), and no significant school x type of acute stressor interaction effect (F_1,182_ = 0.92, p = 0.34). Follow-up ANCOVAs were conducted to evaluate the simple effects of school within type of acute stressor. These analyses, and the data in Figure 1, revealed that noisy-school children had lower SBP reactivity than quiet-school children during both the math (F_1,182_ = 8.53, p = 0.004) and noise challenge (F_1,182_ = 3.95, p = 0.048). School accounted for approximately twice as much variance in SBP reactivity during the math challenge (partial Eta-squared = 0.045) than during the noise challenge (partial Eta-squared = 0.021).

Analyses of DBP reactivity revealed no significant main effect of school (F_1,182_ = 3.46, p = 0.064), no significant main effect of type of acute stressor (F_1,182_ = 2.43, p = 0.121), and no significant school x type of acute stressor interaction effect (F_1,182_ = 1.11, p = 0.29). However, inspection of the means and standard errors in Figure 1 indicated that the schools did differ in DBP reactivity to the math challenge. Thus, follow-up ANCOVAs were conducted to evaluate the simple effects of school within type of acute stressor. These analyses revealed that noisy-school children had significantly lower DBP reactivity than quiet-school children during the math challenge (F_1,182_ = 4.65, p = 0.032), but not during the noise challenge (F_1,182_ = 0.79, p = 0.376). School accounted for a relatively small proportion of the variance (partial Eta-squared = 0.025) in DBP reactivity during the math challenge.

## 4. Discussion

Results of this quasi-experimental study revealed no link between traffic noise exposure at school and resting blood pressure in a sample of urban elementary school boys and girls in India. These findings contradict work on aircraft noise and children’s resting blood pressure, but generally match findings on traffic noise [1,2]. Thus one way to interpret the present results is lack of support for the hypothesis that chronic noise exposure raises blood pressure. However several prior studies have also found that aircraft but not road traffic noise elevates children’s blood pressure. Because we only had two readings to estimate resting blood pressure and there were some potential acute noise effects during the baseline assessments, the data on resting blood pressure and exposure to chronic noise at school are best interpreted as tentative.

The results also did not provide evidence that children exposed to traffic noise at school have different blood pressure responses when they are exposed to acute noise versus non-noise stressors. Instead, children exposed to traffic noise at school appeared to have a generally suppressed blood pressure response to acute stressors relative to children not exposed to traffic noise at school. All analyses controlled for potential confounds, including sex, age, BMI, and problems due to noise and interruptions during testing. The reactivity findings indicate that children from the noisy school tended to have lower blood pressure during exposure to acute noise and non-noise stressors than their counterparts from a quieter school. SBP reactivity was significantly lower in the noisy school than the quiet school during noise and non-noise challenges, whereas DBP reactivity was significantly lower in the noisy school than the quiet school during the non-noise challenge.

To our knowledge, only one other study to date has examined the relation between chronic noise exposure and blood pressure reactivity in children [22], and it found results consistent with the present findings. In that prior study, children in a noisy community near an airport had lower SBP reactivity in response to an acute noise stressor than did their counterparts in a quieter community. Also, the prior study found no relation between noise exposure and DBP reactivity during an acute noise stressor, which also parallels the present findings. As an extension to the prior study, we found that children in a noisy school also had lower SBP and DBP reactivity to a non-noise stressor relative to children attending a quiet school. Thus, there is emerging evidence that chronic noise exposure is linked to dysregulation of blood pressure responses during acute challenges. An insufficient blood pressure response to an acute stressor may reflect an underlying pathophysiological process (e.g., dysregulation of the sympathetic-adrenal-medullary response system) and could undermine effective behavioral responses to stressors that demand a rapid mobilization of energy [26]. The fact that children in the noisy school were less reactive to both acute noise and the psychosocial stressor, mental arithmetic, indicates that physiological desensitization or habituation to noise is an unlikely explanation for the pattern of results uncovered. Rather, the data suggest some general downward shift in stress response efficiency during acute demands.

There are several limitations of this study that must be considered when interpreting these findings. First, because the data were derived from a quasi-experimental design, a number of alternative explanations cannot be ruled out, particularly those related to spurious factors that could be associated with exposure to chronic noise and blood pressure reactivity. For example, community noise tends to covary with other ambient stressors, such as crowding and pollution [26]. Thus it is possible that other factors could have influenced the observed blood pressure differences across schools. Another limitation is that we had insufficient resources for measuring children’s noise exposure outside of school. According to an Environment Status Report by the Pune Municipal Corporation [27], noise levels at most places in the city of Pune exceed recommended levels, ranging from 43 to 109 dB(A). Thus, it is possible that children in both the quiet and noisy school live in communities varying widely in ambient noise levels. This unexplained variance in home noise levels could have attenuated some of the observed relations between noise exposure and blood pressure. A final limitation is that we were less successful at controlling extraneous sources of noise and interruptions in the noisy school than in the quiet school. We were able to add a covariate to control for some of the variance associated with these problems, but it is still possible that variations in the testing environments attenuated some of the effects due to imperfect measurement of ambient conditions during testing at the schools.

## 5. Conclusions

The present results suggest a possible link between chronic noise exposures related to street traffic surrounding elementary schools and diminished blood pressure responses to acute noise and non-noise stressors in elementary-school-aged children. There was no evidence of a link between chronic noise exposure at school and resting blood pressure. In general, data on road traffic noise and blood pressure are equivocal. Since work on aircraft noise and blood pressure is more consistent, at a minimum the noise source as well as its intensity needs to be considered in evaluating the effects of noise on blood pressure. Although not a focus of the present study, we also found that chronic noise exposure at school may be a risk factor for obesity, as evidenced by the significantly higher BMI among children in the noisy versus quiet school. In rapidly developing and highly populous countries, such as India, chronic noise problems will continue to grow exponentially and affect many thousands if not millions of children. Further research is need to establish the long-term and clinical significance of the effects of noise in this context given the vast numbers of people likely to be affected in coming decades.

## Figures and Tables

**Table 1.** Resting systolic and diastolic blood pressure among children in the quiet *versus* noisy elementary school (n = 189).

**Blood Pressure**	**Quiet School**		**Noisy School**			
	***M***	***SE***	***M***	***SD***	***F_df_***	***p***
Systolic 1	94.63	2.00	92.22	2.01	0.56_1,183_	0.452
Diastolic 1	61.71	1.60	56.81	1.62	3.65_1,183_	0.058

1 Means adjusted for sex, age, BMI, and testing problems.

## Abbreviations

SBP: systolic blood pressure
DBP: diastolic blood pressure
mm HG: millimeters of mercury
dB(A): sound pressure using decibel A filter
